# Supplementary material for: A High-Throughput In Vitro Assay for Screening Rice Starch Digestibility
Source: Foods. 2019 Nov 21;8(12):601. doi: 10.3390/foods8120601 (PMC6963981; doi:10.3390/foods8120601)
Supplement: Supplementary file 1 [file foods-08-00601-s001.pdf]

Supplementary Information

Materials and Methods

Materials:
Hydrochloric acid (HCl), potassium hydroxide (KOH), sodium acetate anhydrous (CH_3COONa), and sodium hydroxide pellets (NaOH) and were obtained from Chem-Supply Pty Ltd (Gillman, SA, Australia). Glacial acetic acid (CH_3COOH), magnesium chloride anhydrous ($MgCl_2$) and magnetic stir bars for the total starch assay (Spinkpak®, PTFE-coated, octagonal, 12.7 x 7.9 mm) were sourced from Sigma-Aldrich (Castle Hill, NSW, Australia). Total Starch Assay Kit and D-Glucose Assay Kit (oxidase/ peroxidase, GOPOD format) were sourced from Megazyme International Ireland Ltd. (Wicklow, Leinster, Ireland).

Sample preparation:

Flour samples were prepared by grinding white grains with a hammermill to pass through a 0.5 mm sieve.

Moisture content

The moisture content of rice flour samples was determined by automated thermogravimetric analysis (TGA) using a TGA701 Thermogravimetric Analyser (Leco, Michigan, United States). Each sample was heated under air (dry oil-free) atmosphere up to 130 ℃, ramped at 10.5 ℃ per min until a constant weight was obtained. Moisture analyses were conducted in triplicates, and the results were averaged to obtain a mean value.

Total starch content

Total starch content of rice flour was determined as glucose released by enzymatic hydrolysis after treatment with 2 M KOH using an assay kit (Megazyme Total Starch Assay Kit, Wicklow, Ireland).

Amylose content

Amylose content of flour samples was determined by colorimetric method (iodine binding) as described in AACC International Method number 61-03.01.

Pasting properties

Pasting properties of flour samples were determined with a Rapid Visco Analyser (Perten, Sydney, NSW) by using the protocol described by AACC International Method number 61-02.01.

Gelatinisation Temperature

Gelatinisation temperature was measured using a differential scanning calorimeter (Mettler Toledo model DSC 3) and analysed using STARe software 16.00. The instrument was calibrated with indium, and then the samples were analysed in triplicate on the same day. Flour (5 mg) was mixed with water (15 µL) in hermetic aluminium pans, which were then sealed. The pans were heated under pressure from 45 to 100 °C in increments of 15 °C min–1. The gelatinisation temperature was determined from the peak of the endotherm.

Gel strength

Gel strength was estimated by measuring the firmness value of a gelatinised flour-water slurry. The Stanley-Martin texture test was performed to measure the firmness values using a texture analyser (Perten TVT6700 texture tester) with a 5kg load cell and a cylinder probe (1.6 mm/ s test speed, 20 mm diameter). Rice flour- water slurry samples were prepared at a concentration of 8.3 % (w/w) and heated to 95 ℃ for 12 min. Gels were sealed to prevent moisture loss and stored overnight at 20 ℃ prior to analysis.

Results

Rice geotypes contained low, intermediate and high amylose which was related to other starch characteristics

Prior to starch digestibility assays, eight rice genotypes were analysed for several routine grain quality parameters such as amylose content, thermal properties and cooked rice texture. Table 1 gives the values for total starch (TS), amylose content (AC), peak force (PF) and gelatinisation temperature (GT). TS values were similar for the 8 rice genotypes, between 85.3 % and 87.9 % on a dry weight basis, which were within the ranges previously reported

for rice (Dutta and Mahanta 2012; Zhang et al. 2017). TS content of each sample is required for subsequent analysis of starch digestibility data. According to the AC classification by Juliano (2004) by iodine colorimetry, YRL127 (27.3 % ± 0.9) and Doongara (27.2 % ± 2.0) were high, Waxy (8.2 % ± 0.9) was very low, and the remaining rice genotypes were intermediate, between 20.9 and 21.5 %. The PF parameter, representative of cooked rice hardness, showed a similar trend to AC, whereby PF was highest for YRL127 (305.3 g ± 20.7) and Doongara (260.4 g ± 59.8), lowest for Waxy (25.7 g ± 0.6), and remaining samples were in between (ranging from 92.1 to 129.2 g). The GT for Doongara (74.8 ℃ ± 1.5) and YRL127 (73.6 ℃ ± 2.4) were highest among the eight genotypes, with the remaining samples having slightly lower values, between 67.8 and 70.2 ℃. Table 2 shows the pasting properties of rice genotypes. Lower peak viscosities, lower trough viscosities and higher setback values were observed for the high AC rice genotypes (Doongara and YRL127).

Table 1. Total starch, amylose, peak force and gelatinisation temperature of 8 rice genotypes.

Rice genotype	Total Starch (%) d.w.b.	Amylose (%)	Peak force (g)	Gelatinisation temperature (℃)
Doongara	86.1 ± 1.0	27.2 ± 2.0	260.4 ± 59.8	74.8 ± 1.5
Koshihikari	87.5 ± 1.6	21.5 ± 0.6	129.2 ± 18.4	67.8 ± 0.9
Opus	87.9 ± 1.2	21.3 ± 0.9	112.8 ± 13.4	68.5 ± 0.9
Reiziq	85.3 ± 1.4	20.9 ± 1.1	89.9 ± 16.4	68.2 ± 1.6
Sherpa	85.5 ± 2.1	21.5 ± 1.0	110.3 ± 20.2	68.5 ± 0.9
Topaz	86.5 ± 1.2	21.2 ± 1.1	92.1 ± 21.2	67.9 ± 3.2
Waxy	87.0 ± 1.0	8.2 ± 0.0	25.7 ± 0.6	70.2 ± 0.0
YRL127	85.6 ± 1.4	27.3 ± 0.9	305.3 ± 20.7	73.6 ± 2.4

Table 2. Rapid visco analyser pasting characteristics of starch from 8 rice genotypes.

Rice genotype	Peak viscosity (cP)	Trough viscosity (cP)	Breakdown (cP)	Final viscosity (cP)	Setback (cP)	Pasting temperature (℃)
Doongara	2586	1395	1191	2998	413	74.9
Koshihikari	2984	1674	1310	2903	-81	67.5
Opus	3042	3042	1419	2862	-181	68.5
Reiziq	3058	1859	1198	3132	74	68.7
Sherpa	2850	1735	1115	3043	-181	68.5
Topaz	2894	1557	1337	2956	62	69.5
Waxy	3246	2014	1231	2577	-669	69.9
YRL127	2459	1433	1025	2948	489	73.4

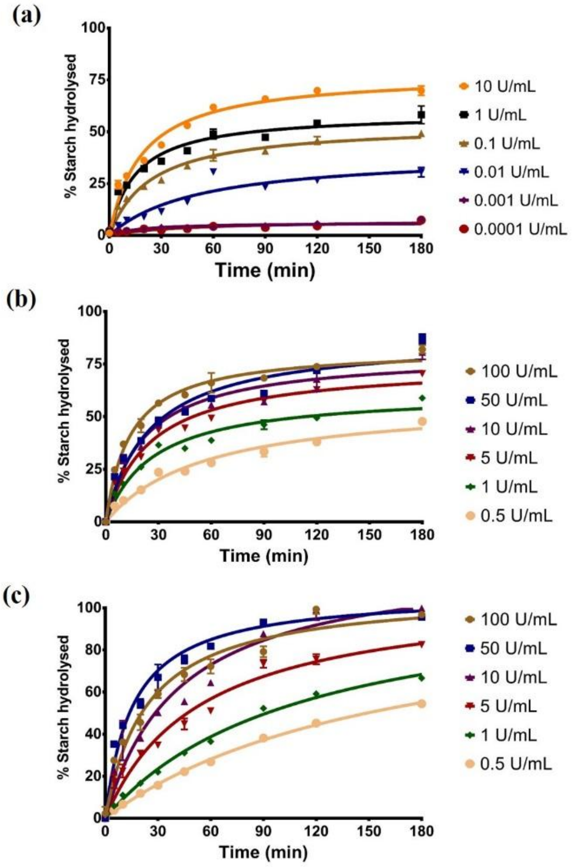

Figure S1. Starch digestograms of cooked Doongara grains using sequential addition of α-amylase (AA) at varying concentrations (0.0001, 0.001, 0.01, 0.1, 1, and 10 U/ mL) with amyloglucosidase (AMG) at 33 U/mL (a), varying concentrations of AMG alone (b) and sequential addition of 1 U/ mL AA with varying concentrations of AMG (b).

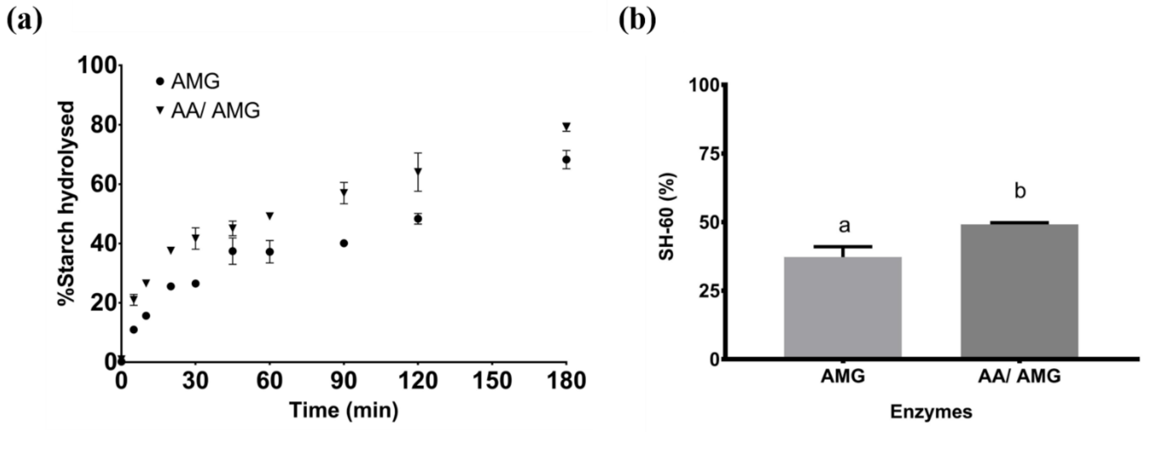

Figure S2. Starch digestogram (a) and starch hydrolysed at 60 min (b) of cooked Doongara grains with sequential addition of α-amylase and amyloglucosidase (AA/ AMG) and amyloglucosidase alone (AMG).

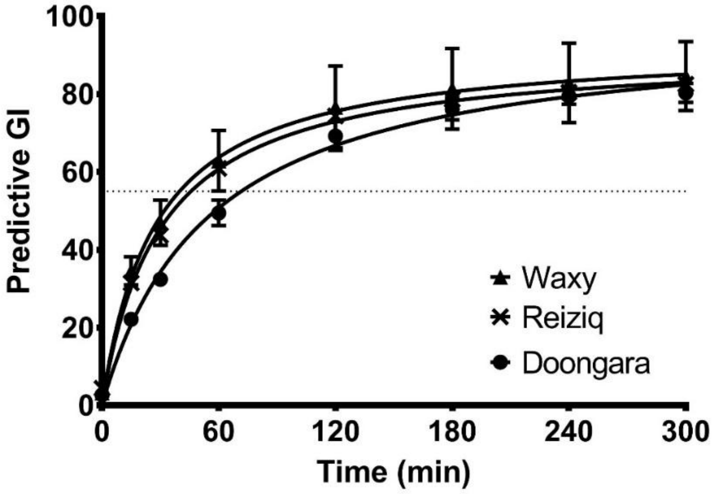

Figure S3. Starch digestogram of three rice genotypes (n = 3) measured by commercially available instrument. The dotted line denotes 55 % of starch hydrolysed.

References

Dutta, H. and Mahanta, C. L. 2012. Effect of hydrothermal treatment varying in time and pressure on the properties of parboiled rices with different amylose content. Food Research International 49:655-663.

Juliano, B. O. 2004. Rice: Overview. Pages 41-48 in: Encyclopedia of Grain Science, 1st. C. Wrigley, ed. Elsevier: Oxford.

Zhang, C. Q., Chen, S. J., Ren, X. Y., Lu, Y., Liu, D. R., Ca, X. L., Li, Q. F., Gao, J. P. and Liu, Q. Q. 2017. Molecular structure and physicochemical properties of starches from rice with different amylose contents resulting from modification of OsGBSSI activity. Journal of agricultural and food chemistry 65:2222-2232.
